# Current pain management practices for preterm infants with necrotizing enterocolitis: a European survey

**DOI:** 10.1038/s41390-023-02508-2

**Published:** 2023-02-24

**Authors:** Judith A. ten Barge, Gerbrich E. van den Bosch, Naomi J. Meesters, Karel Allegaert, Cristina Arribas, Giacomo Cavallaro, Felipe Garrido, Genny Raffaeli, Marijn J. Vermeulen, Sinno H. P. Simons, on behalf of the ESPR Special Interest Group for Neonatal Pain and the NEC Pain Study Group

**Affiliations:** 1 Department of Pediatrics, Division of Neonatology, Sophia Children’s Hospital, Rotterdam, The Netherlands; 2 Department of Development and Regeneration, KU Leuven, Leuven, Belgium; 3 Department of Pharmaceutical and Pharmacological Sciences, KU Leuven, Leuven, Belgium; 4 Department of Hospital Pharmacy, Erasmus MC, Rotterdam, The Netherlands; 5 Department of Pediatrics, Clínica Universidad de Navarra, Madrid, Spain; 6 Neonatal Intensive Care Unit, Fondazione IRCCS Ca’ Granda Ospedale Maggiore Policlinico, Milan, Italy; 7 Department of Clinical Sciences and Community Health, Università degli Studi di Milano, Milan, Italy

## Abstract

**Background:**

Necrotizing enterocolitis (NEC) is a highly painful intestinal complication in preterm infants that requires adequate pain management to prevent short- and long-term effects of neonatal pain. There is a lack of international guidelines for pain management in NEC patients. Therefore, this study aims to describe current pain management for NEC patients in European neonatal intensive care units (NICUs).

**Methods:**

An online survey was designed and conducted to assess current practices in pain management for NEC patients in European NICUs. The survey was distributed via neonatal societies, digital platforms, and professional contacts.

**Results:**

Out of the 259 responding unique European NICUs from 36 countries, 61% had a standard protocol for analgesic therapy, 73% assessed pain during NEC, and 92% treated NEC patients with intravenous analgosedatives. There was strong heterogeneity in the used pain scales and initial analgesic therapy, which mainly included acetaminophen (70%), fentanyl (56%), and/or morphine (49%). A third of NICU representatives considered their pain assessment adequate, and half considered their analgesic therapy adequate for NEC patients.

**Conclusions:**

Various pain scales and analgesics are used to treat NEC patients in European NICUs. Our results provide the first step towards an international guideline to improve pain management for NEC patients.

**Impact:**

- This study provides an overview of current pain management practices for infants with necrotizing enterocolitis (NEC) in European neonatal intensive care units.
- Choice of pain assessment tools, analgosedatives, and dosages vary considerably among NICUs and countries.
- A third of NICU representatives were satisfied with their current pain assessment practices and half of NICU representatives with their analgesic therapy practices in NEC patients in their NICU.
- The results of this survey may provide a first step towards developing a European pain management consensus guideline for patients with NEC.

## Introduction

Necrotizing enterocolitis (NEC) is a serious and very painful inflammatory bowel condition mainly affecting preterm neonates, with a prevalence of 5–10% among very-low-birth-weight infants (VLBW, <1500 g) and a mortality ranging from 15 to 50%.^1,2^ Although the etiology of NEC has not been fully unraveled yet, intestinal immaturity, enteral feeding, the intestinal microbiome, inflammation, and local ischemia are involved.^3^ The excessive intestinal inflammation that characterizes NEC may affect distant organs such as the brain, thereby predisposing survivors of NEC to neurodevelopmental disability.^4,5^ Moreover, the excessive inflammation and ischemia in the intestine cause severe visceral pain.^6^

Optimal pain treatment in patients with NEC is not only needed to protect against the burden of pain, but also to promote recovery and outcome. Exposure to pain during the neonatal period has been associated with negative short-term effects, such as increased circulatory and metabolic complications.^7^ Described long-term effects of neonatal pain include changes in brain morphology,^8–13^ altered pain sensitivity,^14–20^ and impaired cognitive development and behavior.^21–25^ Data and studies on pain management for patients with NEC are largely lacking. In the Netherlands, acetaminophen and opioids such as morphine and fentanyl are most commonly used for treating pain in patients with NEC.^26,27^ A retrospective study has shown that despite analgesic therapy during NEC, most patients still experienced episodes of pain and a quarter of patients experienced persisting periods of pain, with a median duration of 7 h.^27^ This observation suggests that current pain management in patients with NEC may be inadequate. Since pain management is based on pain assessment, (non-)pharmacological therapy and subsequent re-assessment, inadequate pain management reflects deficiencies in either one or a combination of these steps.

To our knowledge, international guidelines for pain management for patients with NEC have not been developed yet. Therefore, pain management practices for patients with NEC among European neonatal intensive care units (NICUs) may vary considerably. Insight into pain management practices across Europe and possible consensus therein may contribute to identifying the optimal pain management strategy for patients with NEC and may thereby improve their outcomes. Therefore, this study aims to describe current pain management practices for patients with NEC in European NICUs, including pain assessment and (non-)pharmacological therapy, with the ultimate goal to develop European consensus guidelines for pain management in patients with NEC.

## Methods

### Study design and participants

A cross-sectional study was conducted to assess pain management practices (i.e., pain assessment and analgesic therapy) across European NICUs via a web-based survey. The head of the NICU or another senior neonatologist was requested to complete the survey. This study aimed to include one representative per European NICU, although there were no restrictions, because the survey was open-access. This study was deemed exempt from ethical approval, due to the voluntary and anonymous nature of the physicians’ responses. Participants were informed that submitting the survey meant agreeing to participate.

### Survey

The survey was developed in LimeSurvey version 2.06 (LimeSurvey GmbH, Hamburg, Germany). The included items were generated based on a literature review and the expert opinion of the authors. The validity of the content of the survey was ensured in a two-step process. Firstly, the survey was pre-tested by NICU clinicians working at the Erasmus MC—Sophia Children’s Hospital and subsequently revised based on their feedback. Secondly, an international group of eight neonatologists and a nurse specialist, all having expertise in pain management and/or survey research, evaluated the content validity of the survey by assessing its relevance, comprehensiveness, and comprehensibility. Based on their feedback, six questions were added, one answer option was added, and one question was rephrased.

The final version of the survey included 39 questions about NICU demographics, protocols for analgesic therapy, pain assessment, non-pharmacological interventions, use of intravenous analgesics, dosing regimen, and the respondent’s expert opinion on current pain management in his/her NICU (Supplementary Material 2).

The survey was distributed through national neonatology societies, the European Society for Pediatric Research (ESPR), 99NICU, LinkedIn, and professional contacts. The survey was open from November 25, 2021, until February 3, 2022. In order to maximize the response rate, reminders were sent to contacts in countries with a low response rate.

### Statistical analyses

All submitted responses were checked for duplicates from the same center. Only responses from unique European centers were included in the primary analysis. In case of more than one response per NICU, only the first response was included in the primary analysis. Non-European responses and duplicate responses were included in secondary analyses.

Descriptive statistics have been presented as median (interquartile range) or number (percentage), depending on the type of data. Analyses were conducted with RStudio version 2021.09.2 and R version 4.1.2 (R Core Team, Vienna, Austria). In order to prevent distortion of the results by typographical errors, respondents whose dose response deviated at least fourfold from the median dose of that analgesic were contacted to verify their response. Response percentages per country were calculated by dividing the number of responses from a country by the total number of NICUs in that country, as indicated by professional contacts or online sources.

To evaluate whether the response of one neonatologist is a reliable measure of pain management practices in the NICU, we assessed the consistency between the numerical and multiple choice responses of duplicate responses from NICUs. Within-NICU agreement was evaluated by calculating the Intraclass Correlation Coefficient (ICC) for numerical questions, based on an average measures, absolute-agreement, two-way random-effects model, and Fleiss’ Kappa statistic for multiple choice questions.^28,29^ Within-NICU agreement was summarized by calculating the median (IQR) of the ICC’s and Fleiss’ Kappa statistics per NICU.

Responses to the final open question, regarding the respondent’s suggestions for improvement of pain management for patients with NEC, were analyzed with thematic content analysis, using the approach described by Braun and Clarke.^30^ Responses were coded with NVivo version 1.6.2 (QSR International Pty Ltd, Melbourne, Australia).

## Results

### Responding NICUs

**Fig. 1:**
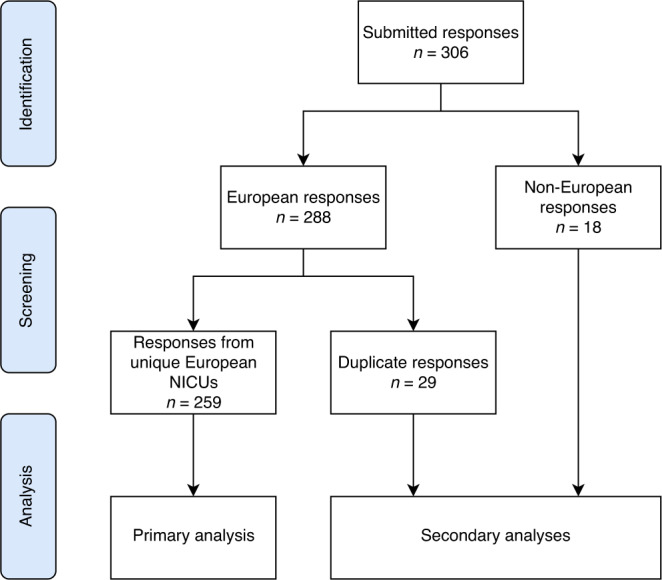
Flowchart of the inclusion and analysis of survey responses. Responses from unique European NICUs were included in the primary analysis. In case of multiple responses per NICU, the first one was included in the primary analysis. Duplicate responses and responses from NICUs outside Europe were included in secondary analyses.

A total number of 306 responses were submitted, of which 288 originated from European NICUs and 18 from non-European NICUs. Figure 1 shows the flowchart of the inclusion and analysis of responses. The responses from European NICUs included 259 responses from unique NICUs and 29 duplicate responses. Table 1 shows the background characteristics of the unique European NICUs. Sixty-three percent of NICU representatives characterized the level of care provided by their NICU as level IV. The median number of annual neonatal admissions to the NICU was 400 (IQR 250–600), including a median number of 65 (IQR 39–120) VLBW admissions. Over half of the NICUs treated 1–10 patients with NEC yearly and approximately a third treated more than 10 patients with NEC yearly. Fifteen NICUs did not treat any patients with NEC yearly and therefore received no further questions about pain management for patients with NEC.

**Table 1:** Background characteristics of the responding unique European NICUs (*n* = 259).

Variable	
Level of care provided by NICUs	
Level I	2 (0.8)
Level II	23 (8.9)
Level III	85 (32.8)
Level IV	162 (62.5)
Function of respondent	
Neonatologist	243 (93.8)
Pediatrician	4 (1.5)
Other	12 (4.6)
Number of neonatal admissions in 2020	400 (250–600)
Number of VLBW neonatal admissions in 2020	65 (39–120)
Number of NEC patients treated in the NICU yearly	
0	15 (5.8)
1–10	150 (57.9)
10–20	56 (21.6)
20–30	15 (5.8)
30–40	8 (3.1)
40–50	5 (1.9)
>50	3 (1.2)
Unknown	7 (2.7)

Values are expressed as median (IQR) or number (%).

**Fig. 2:**
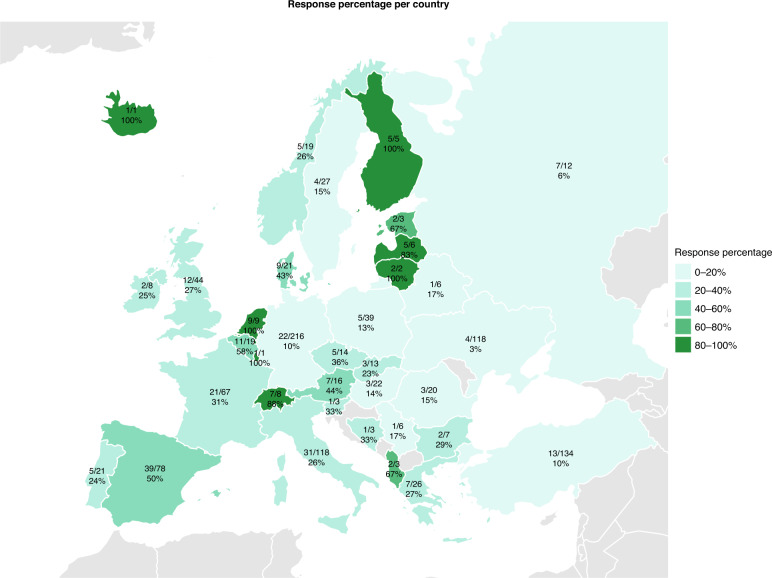
Map displaying the number of responses, total number of NICUs and response percentage per country. The response percentage per country varied from 3 to 100% and the overall response percentage was 21%.

NICU representatives from 36 out of 44 European countries (82%) responded to the survey. Figure 2 shows a map of the number of unique responses and the response percentage per European country based on the total number of NICUs in each country. The overall response percentage was 21% and the response percentage per country ranged from 3 to 100%.

### Protocols for pain management

Out of the 259 unique European NICUs, 158 NICUs (61%) had a written protocol for analgesic therapy in neonates. In 64% of these NICUs, this protocol included clear definitions for starting, stopping, and adjusting analgesics. Only 7% of NICUs with a protocol for analgesic therapy (4% of all responding NICUs) had a specific NEC pain management protocol. The level of adherence to the protocol, as rated by the respondent, was very high in 25% of NICUs, high in 43%, intermediate in 27%, and low in 5%.

### Pain assessment

**Fig. 3:**
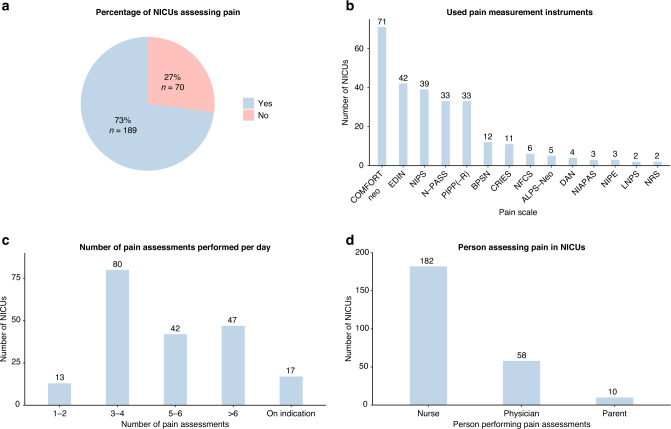
Pain assessment in European NICUs. Pie chart displaying the percentage of NICUs assessing pain in patients with NEC (**a**), and bar charts displaying the used pain measurement instruments (**b**), the number of pain assessments (**c**), and the performer of pain assessments (**d**).

Seventy-three percent of NICUs assessed pain levels in patients with NEC. Figure 3 shows an overview of pain assessment practices in European NICUs, including used measurement instruments, assessor, and frequency of assessments. The most commonly used pain measurement instruments were the COMFORTneo score (38% of NICUs assessing pain), Échelle de la Douleur Inconfort Nouveau-Né (EDIN) (22%), Neonatal Infant Pain Scale (NIPS) (21%), Neonatal Pain, Agitation and Sedation Scale (N-PASS) (18%), and Premature Infant Pain Profile (PIPP) (18%). Supplementary Material 3 shows the most used pain measurement instrument per country. Twenty-eight NICUs (15%) reported using multiple pain measurement instruments. Almost all NICUs solely relied on behavioral pain measurement instruments, with only three NICUs (also) using Newborn Infant Parasympathetic Evaluation (NIPE) to determine pain levels. The pain assessors included nurses in 96% of NICUs, physicians in 31%, and parents in 5.3%. The number of pain assessments per day was 1–2 in 6.9% of NICUs, 3–4 in 42%, 5–6 in 22%, >6 in 25%, and/or on indication in 9.0%.

### Pain management practices

Non-pharmacological interventions, sucrose, intravenous analgesics, and epidural analgesics were used in 58%, 39%, 92%, and 2% of NICUs treating patients with NEC, respectively.

#### Non-pharmacological

In addition to sucrose, the following non-pharmacological interventions were most commonly reported: containment (e.g., facilitated tucking, swaddling) in 35% of NICUs, touch (e.g., holding, kangaroo care) in 18%, non-nutritive sucking in 18%, positioning in 9%, and parental involvement in 5%. Supplementary Material 4 shows an overview of pain management practices for patients with NEC.

#### Pharmacological

Out of the 225 NICUs using intravenous analgesics for patients with NEC, 53 NICUs (24%) initiated analgesic therapy pre-emptively. The most common moments to initiate pre-emptive analgesic therapy were after diagnosis of NEC stage ≥II (70%) or surgery (45%). In 44% of NICUs, analgesic therapy was different for ventilated versus spontaneously breathing patients with NEC. NICU representatives mostly commented that in those who breath spontaneously, lower opioid doses are used or that opioids are (preferably) avoided. In 11% of NICUs using intravenous analgesics for patients with NEC, certain analgosedatives were classified as contra-indicated, such as midazolam (3.1%), morphine (2.2%), ibuprofen (1.8%), propofol (1.3%), and fentanyl (1.3%).

**Fig. 4:**
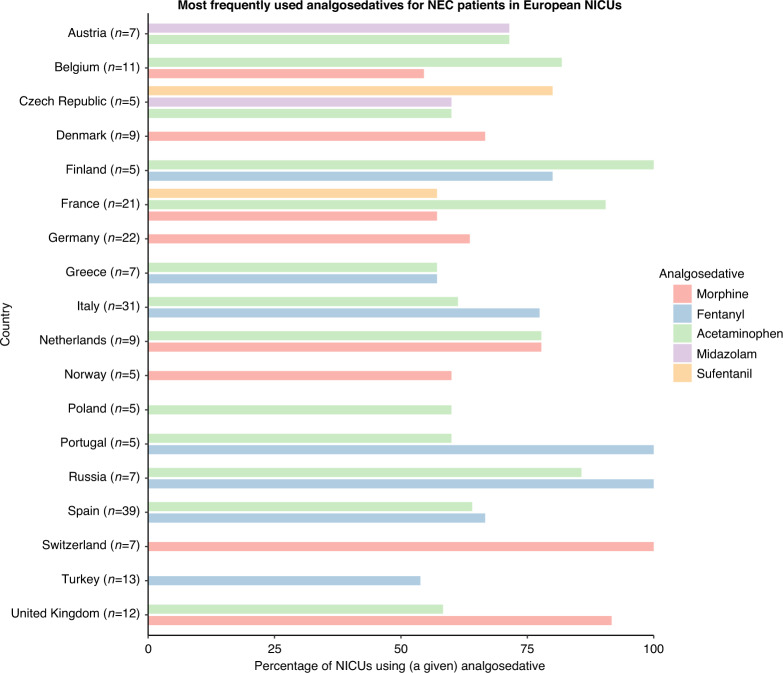
Most frequently used analgosedatives for patients with NEC per country. Only countries with five or more responses are included. Only analgosedatives that are used in more than half of the NICUs in the country are included.

The most commonly used analgosedatives for initial analgesic therapy were acetaminophen (70% of NICUs using intravenous analgesics), fentanyl (56%), morphine (49%), midazolam (25%), sufentanil (11%), and ketamine (6.7%). Acetaminophen, fentanyl, and/or morphine were often (38% of NICUs using intravenous analgesics) used in combinations of two or three of these analgesics. The use of analgosedatives varied across countries, with some countries mainly using fentanyl and others mainly using acetaminophen or morphine (Fig. 4). Supplementary Material 5 shows the most used analgosedative per country. As shown in Table 2, acetaminophen was exclusively administered intermittently; fentanyl and morphine were mainly administered continuously plus intermittently; and midazolam and sufentanil were mainly administered continuously.

**Table 2:** First choice intravenous analgosedatives for pain treatment in NEC patients and type of administration per analgosedative (*n* = 225 NICUs).

Analgosedative	Total	Continuous administration	Intermittent administration	Continuous + Intermittent	Loading dose used
Acetaminophen	157 (69.8)	0 (0.0)	157 (100.0)	0 (0.0)	0 (0.0)
Fentanyl	125 (55.6)	56 (44.8)	11 (8.8)	58 (46.4)	30 (26.5)
Morphine	111 (49.3)	39 (35.1)	13 (11.7)	59 (53.2)	61 (62.2)
Midazolam	57 (25.3)	33 (57.9)	5 (8.8)	19 (33.3)	16 (30.8)
Sufentanil	25 (11.1)	16 (64.0)	1 (4.0)	8 (32.0)	6 (25.0)
Ketamine	15 (6.7)	4 (26.7)	3 (20.0)	8 (53.3)	4 (33.3)
Nalbuphine	5 (2.2)	0 (0.0)	3 (60.0)	2 (40.0)	1 (50.0)
Tramadol	4 (1.8)	1 (25.0)	2 (50.0)	1 (25.0)	1 (50.0)
Clonidine	4 (1.8)	4 (100.0)	0 (0.0)	0 (0.0)	2 (50.0)
Dexmedetomidine	4 (1.8)	4 (100.0)	0 (0.0)	0 (0.0)	0 (0.0)
Methadone	3 (1.3)	0 (0.0)	3 (100.0)	0 (0.0)	0 (0.0)
Remifentanil	3 (1.3)	3 (100.0)	0 (0.0)	0 (0.0)	0 (0.0)
Metamizole	2 (0.9)	0 (0.0)	2 (100.0)	0 (0.0)	0 (0.0)
Oxycodone	1 (0.4)	0 (0.0)	1 (100.0)	0 (0.0)	0 (0.0)
Ketofol	1 (0.4)	0 (0.0)	0 (0.0)	1 (100.0)	1 (100.0)
Piritramide	1 (0.4)	0 (0.0)	1 (100.0)	0 (0.0)	0 (0.0)
Pethidine	1 (0.4)	0 (0.0)	1 (100.0)	0 (0.0)	0 (0.0)

Values are expressed as number of NICUs (%).

**Fig. 5:**
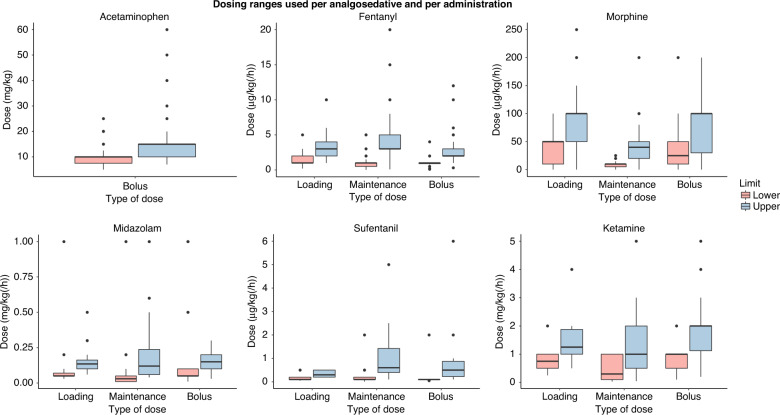
Dose ranges used per analgosedative and per administration route for the six most commonly used analgosedatives. There were 22 extreme outliers in the submitted midazolam doses, ranging from 2 to 200 mg/kg(/h), which were excluded from this boxplot.

Figure 5 shows the prescribed dose ranges for the most commonly used analgosedatives. There seemed to be more consensus on the used starting doses (i.e., lower limits) than the maximum doses (i.e., upper limits). An overview of prescribed dose ranges for all analgosedatives is shown in Supplementary Material 6.

**Fig. 6:**
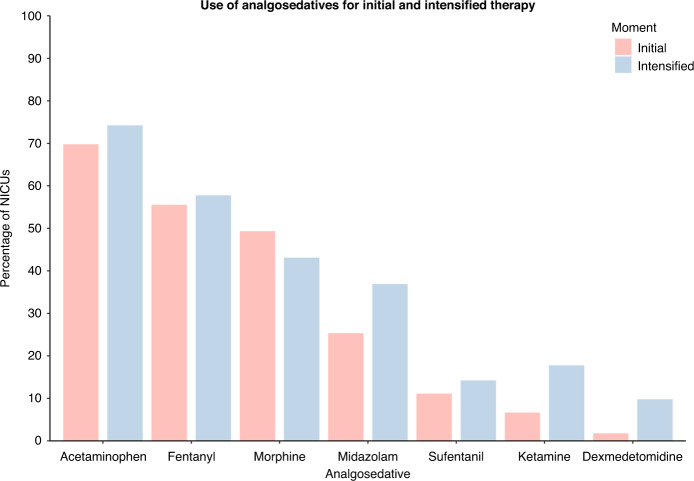
Use of analgosedatives for initial and intensified analgesic therapy. Acetaminophen, fentanyl, midazolam, sufentanil, ketamine, and dexmedetomidine were mainly used for intensified analgesic therapy, whereas morphine was mainly used as initial treatment option.

In case of pain despite initially started analgesic therapy, 207 NICUs (92%) intensified analgesic therapy by increasing the dose of the current analgosedatives, 120 NICUs (53%) by adding another analgosedative, and 51 NICUs (23%) by switching to other analgosedatives. NICUs that chose to increase the dose most commonly increased the dose of fentanyl (49%) or morphine (42%). The most commonly added analgosedatives were midazolam (13%), ketamine (11%), dexmedetomidine (7.6%), fentanyl (7.1%), and acetaminophen (7.1%). Figure 6 shows the most frequently used analgosedatives for initial and intensified analgesic therapy. Supplementary Material 7 shows an overview of intensified analgesic therapy strategies.

### Pain management in NICUs outside Europe

Another total of 18 neonatologists working in NICUs in 14 non-European countries completed the survey. Supplementary Material 8 shows the number of responses per non-European country. Four NICUs (22%) had a written standard protocol for analgesic therapy, nine (50%) assessed pain in patients with NEC, and 14 (78%) administered intravenous analgesic therapy to patients with NEC. The NIPS was the most widely used scale among non-European NICUs, with five NICUs using this scale. Fentanyl was the most commonly used analgesic for initial therapy (ten NICUs), followed by acetaminophen (eight NICUs), morphine (four NICUs), midazolam (three NICUs), and dexmedetomidine (one NICU).

### Agreement between duplicate responses per NICU

There were 23 cases in which two neonatologists from the same NICU submitted the survey and three cases in which three neonatologists from the same NICU submitted the survey, resulting in a total of 29 duplicate (i.e., non-unique) responses. In general, agreement between responses from different respondents working in the same NICU was good to excellent, with a median Intraclass Correlation Coefficient of 0.899 (IQR 0.865–0.974) for the numerical answers.^29^ The median Fleiss’ Kappa statistic was 0.690 (IQR 0.604-0.742), indicating fair to good agreement in the multiple choice answers.^31^

### Opinions on pain management in their NICU

Approximately a third of NICU representatives considered their locally used pain measurement instrument adequate in patients with NEC. Half of the NICU representatives considered their analgesic therapy regimen adequate for patients with NEC.

Three major themes were identified in NICU representatives’ responses to the open question on suggestions to improve pain management for patients with NEC. (1) Pain assessment: Of those with suggestions (*n* = 159), 62 NICU representatives (39%) identified pain assessment as a target to improve pain management, with 40 of them mentioning that better tools for pain assessment are needed (e.g., incorporating physiological parameters) and 12 mentioning that pain should be assessed more frequently. (2) Pain protocols: Forty-nine NICU representatives (31%) expressed a need for more standardization in pain management for patients with NEC by establishing protocols/guidelines. Eleven of them remarked that these protocols should be specific for NEC, and four remarked that they should be evidence-based. (3) Analgesic therapy: According to 43 NICU representatives (27%), better analgesic therapy was needed to improve pain management for patients with NEC. This most commonly entailed using another analgesic (*n* = 25), administering pre-emptive analgesic therapy (*n* = 6), or “more aggressive” analgesic therapy (*n* = 5). Out of those preferring to use another analgesic, a few mentioned a specific agent, but the majority mentioned that new drugs are needed which are more effective and/or have fewer adverse effects. Additional common themes included: need for more research, education of staff, awareness, non-pharmacological interventions, parental involvement, and teamwork.

## Discussion

This European cross-sectional survey study on current pain management practices for preterm infants with NEC showed that there is a large variability in pain management practices, including the used pain measurement instruments and choice and dose ranges of analgosedatives. Furthermore, we showed that only a third of NICU representatives consider their current pain assessment adequate and half of them consider their analgesic therapy adequate. Three-quarters of the responding NICUs assessed pain levels in patients with NEC and almost all NICUs provided intravenous analgesic therapy, which was protocolized in 60% of the NICUs. Analgesic therapy most commonly included acetaminophen, fentanyl and/or morphine. In case of pain under initial analgesic therapy, analgesic therapy was most commonly intensified by increasing the dose of the current analgosedatives (e.g., fentanyl or morphine) or adding another analgosedative (e.g., midazolam or ketamine). The maximum used doses of analgosedatives varied considerably between NICUs, suggesting that currently at least some NEC patients may receive excessive or insufficient dosages of analgosedatives.

To our knowledge, pain management for patients with NEC in different NICUs has not been studied before in Europe nor in other parts of the world. A previous study which assessed overall pain management practices (pain assessment and analgesic therapy) in Europe focused on the general NICU population, not on NEC patients specifically.^32,33^ Regarding pain assessment, this EUROPAIN study found that half of the included NICUs ever performed assessments of prolonged pain.^33^ Furthermore, the EUROPAIN study found that the EDIN scale (57%) was most commonly used, followed by the COMFORTneo scale (20%) and the N-PASS (13%). Our study found a larger proportion of NICUs assessing pain in NEC patients (73%), although unlike the EUROPAIN study, we did not specify that a pain measurement instrument designed for prolonged pain assessment had to be used. Two of the most used instruments reported in our study, the NIPS and the PIPP, have been validated for assessing procedural pain, not prolonged pain.^34–36^ The COMFORTneo, EDIN and N-PASS have been validated for assessing prolonged pain in infants, although not specifically for use in patients with NEC.^37–39^ Behavioral pain scales may be less valid in patients with NEC, since these patients are known to exhibit few movements and diminished facial expression due to their clinical condition.^37^ Instruments incorporating physiological parameters, such as NIPE, might provide a solution. However, studies evaluating the validity of NIPE are scarce and report contradictory results.^40^

Regarding analgesic therapy, the EUROPAIN study found that acetaminophen, morphine, fentanyl, and midazolam were the most used analgosedatives.^32^ Our study shows that these medications are also commonly used in patients with NEC. In addition to these medications, we found that sufentanil, ketamine, and dexmedetomidine are frequently used, especially for intensified therapy. This confirms the observation by Stark et al that dexmedetomidine is increasingly being used in neonatal care.^41^ Our study extends the EUROPAIN study by not only assessing choice of analgosedatives, but also strategies for intensified analgesic therapy and dose ranges used. We found that the maximum used doses of morphine, fentanyl, and midazolam varied considerably. In over a quarter of NICUs using these analgesics, the used maximum maintenance doses of morphine and fentanyl exceeded the dosing ranges recommended in a consensus statement for neonatal pain management.^42^ The reported midazolam maintenance doses varied over a thousand fold range, likely reflecting a mix-up between microgram and milligram in the outlier responses.

A major strength of our study is the coverage of over 250 NICUs from 36 countries, resulting in an extensive overview of pain management for patients with NEC across Europe. However, the response percentage varied significantly between countries, which may partly be explained by the high number of smaller NICUs in some countries. It cannot be ruled out that NICUs with an interest in pain management were more likely to respond to the survey, thereby causing selection bias. For feasibility reasons, only one neonatologist per NICU was requested to complete the survey. Targeting the head of the NICU or another senior neonatologist may have resulted in exaggerated satisfaction with current practices in comparison with junior neonatologists and nurses. Moreover, the response of one neonatologist may not have been representative for practices in the NICU, since practices and interpretation of guidelines may differ among neonatologists working in the same NICU. However, evaluation of the consistency between responses of different neonatologists working in the same NICU showed good agreement within NICUs.

The results of this study provide a first step towards improvement of pain management for patients with NEC, which is crucial since (severe) pain is common in preterm infants with NEC.^27^ Inadequate analgesia may hamper the recovery from NEC, and exposure to pain during the neonatal period is associated with negative short-term and long-term effects, including altered brain development.^43^ Currently, there seems to be consensus on the necessity of performing pain assessments and administering analgesic therapy, but limited consensus on choice of pain measurement instruments, analgesics, and dosages. The large variety in pain management practices suggests that currently some patients with NEC may receive excessive or insufficient treatment, leaving them vulnerable to negative consequences of exposure to pain or excessive use of opioids or other potentially toxic drugs. In order to establish good pain management guidelines for patients with NEC, future studies are needed on effectiveness and safety of different analgesic therapy regimens. Meanwhile, a debate among neonatal pain experts is necessary to reach a consensus on pain management recommendations, including pain assessment tools and analgesic algorithms, using for example the Delphi method.^44^

## Conclusions

There is large variability in the use of pain protocols, pain scales and analgesic therapies for preterm infants with NEC across European NICUs. Data from the current study may provide the basis to develop consensus pain management guidelines and highlight opportunities to improve pain management for preterm infants with NEC.

## Supplementary Information

Supplementary Material 1

## Data Availability

The datasets generated and analyzed during the current study are available from the corresponding author on reasonable request.
