# Supplementary material for: Current pain management practices for preterm infants with necrotizing enterocolitis: a European survey
Source: Pediatr Res. 2023 Feb 24;94(2):555–63. doi: 10.1038/s41390-023-02508-2 (PMC10382315; doi:10.1038/s41390-023-02508-2)

Supplementary Material 1. Members of the ESPR Special Interest Group for Neonatal Pain and the NEC Pain Study Group

European Society for Paediatric Research (ESPR) Special Interest Group for Neonatal Pain Members

Agnes van den Hoogen
Angela Amigoni
Anne Smits
Aomesh Bhatt
Bert Joosten
Caroline Ahearne
Charalampos Kotidis
Charles Roehr
Christ-Jan van Ganzewinkel
Cristina Arribas
Daniëlla Roofthooft
Elisabeth Norman
Emma Olsson
Eugene Dempsey
Felipe Garrido
Fransesca Sperotto
Genny Raffaeli
Gerbrich van den Bosch
Giacomo Cavallaro
Helle Lønstrup Haslund-Thomsen
Jannicke Andresen
Jean-Michel Roué
Joke Wielenga
Judith ten Barge
Karel Allegaert
Katrin Klebermass-Schrehof
Laura Moschino
Liam Mahoney
Luke Baxter
Manon Tauzin
Mats Eriksson
Naomi Meesters
Paola Lago
Rebeccah Slater
Ricardo Carbajal
Robert Flint
Serdar Beken
Sezin Ünal
Sinno Simons
Solfrid Steinnes
Swantje Völler
Xavier Durrmeyer

List of participants in the survey (NEC Pain Study Group). Only participants who wished to provide their names and affiliations have been included.

Agnese, Fondazione Ca Granda Policlinico Milano (Italy)
Agnieszka Kordek, Pomeranian Medical University in Szczecin (Poland)
Agnieszka Kordek, Pomeranian Medical University in Szczecin (Poland)
Agustin Bernatzky, Sanatorio Anchorena San Martin (Argentina)
Alain Beuchée, CHU Rennes (France)
Alejandro López Escobar, Hospital Vithas Madrid La Milagrosa (Spain)
Alexander Obedin, Gbuz sk sccpc1 stavropol region (Russia)
Alexandre Lapillonne, Necker Enfants Malades University hospital, Paris (France)
Ali Abdelkader Abdelah, Hospital Comarcal de Melilla (Spain)
Alice Proto, GOM Niguarda, Milan (Italy)
Aline Vuckovic, Hôpital Universitaire des Enfants Reine Fabiola (Belgium)
Amitava Sur, Lancashire Women and Newborn Center (United Kingdom)
Ana María Baña Souto, Clínico Santiago (Spain)
Ana Vidal Esteban, Hospital Universitario de Móstoles (Spain)

André Léké, CHU Amiens (France)
Andre M Graca, Centro Hospitalar Universitario de Lisboa Norte (Portugal)
Andrea Dotta, Bambino Gesù Children's Hospital Rome (Italy)
Andreas Jenke, Klinikum Kassel (Germany)
Andreas Mueller, Children's Hospital, University of Bonn (Germany)
Andreas W. Flemmer, LMU University Hospital (Germany)
Aneta Soltirovska Salamon, University Medical Center, Ljubljana (Slovenia)
Anita Dumitrescu, Vivantes Klinikum Friedrichshain (Germany)
Anna Onnela, Saint-Luc University hospital (Belgium)
Anna Vinaixa, Hospital Dexeus (Spain)
Anne-Cathrine Viuff, Aalborg University Hospital (Denmark)
Arantxa Vidal Esteban, Hospital Universitario de Fuenlabrada (Spain)
Artem Chernykh, Kemerovo State Pediatric Clinical Hospital (Russia)
Arturo Hernández de Bonis, Hospital Universitario de Toledo (Spain)
Asunción Pino Vázquez, Clinico Universitario De Valladolid (Spain)
Axel Franz, University Tübingen (Germany)
Bachy Antoine, GHDC Charleroi (Belgium)
Barbara Królak-Olejnik, Medical University in Wrocław (Poland)
Beata Łoniewska, Pomeranian Medical University in Szczecin (Poland)
Beatriz Vacas del Arco, Hospital Universitario de Salamanca (Spain)
Beba Kalkan, Paediatric Hospital (Bosnia and Herzegovina)
Belen Fernandez Colomer, Hospital Universitario Central de Asturias (Spain)
Belma Karagöl, Gülhane Medicine Faculty (Turkey)
Benjamim Ficial, Azienda Ospedaliera Universitaria Integrata Verona (Italy)
Bernhard Bungert, Klinikum Hanau (Germany)
Bin Huey Quek, KK Women's and Children's Hospital (Singapore)
Boeuf, CHU de Clermont Ferrand (France)
Borenstein-Levin Liron, Rambam Medical Center (Israel)
C.E. Schwarz, Children's University Hospital Tübingen (Germany)
Caridad Tapia Collados, Hospital General Universitario Alicante (Spain)
Carlos Manuel Perez Valdez, Hospital el Pilar (Guatemala)
Carmen Carvalho, Centro Materno Infantil do Norte (Portugal)
Celia Fabra Garrido, La Paz University Hospital (Spain)
Charles Roehr, Southmead Hospital, North Bristol Trust, Bristol (United Kingdom)
Chatzakis Emmanouil, Venizelio General Hospital (Greece)
Chris Gale, Chelsea and Westminster Hospital (United Kingdom)
Christoph Berger, LKH Hochsteiermark (Austria)
Christoph Binder, Medical University Vienna (Austria)
Colm O'Donnell, National Maternity Hospital & University College Dublin (Ireland)
Consuelo Vazquez Gomis, HGU Elche (Spain)
Corinne Däster, Kantonsspital Aarau (Switzerland)
Cristina Encabo Gil, Hospital de Valme (Spain)
Dace Sniedze, BKUS Riga (Latvia)
Dana Dolníková, National istitute of children’s disease (Slovakia)
Daniel Vijlbrief, UMC Utrecht (Netherlands)
David Sweet, Royal Maternity Hospital, Belfast (United Kingdom)
Deniz Anuk Ince, Baskent University Hospital (Turkey)
Denys Surkov, Dnipro Regional Children's Hospital (Ukraine)
Dilek Kahvecioğlu, University of Health Sciences Ankara Training and Research Hospital (Turkey)
Dina Apele-Freimane, Pauls Stradins Clinical University Hospital (Latvia)
Dominique Singer, University Medical Center Hamburg-Eppendorf (Germany)
Dussart Anneliese, CHU Tivoli (Belgium)
DWE Roofthooft, Erasmus MC (Netherlands)
Dzmitry Sankovec, National Research and Practical Center Mother and Child (Belarus)
E. Motte-Signoret, CHI Poissy (France)
Edmondo N. L. Hammond, Diakonie Krankenhaus Bad Kreuznach (Germany)
Eduard Lobera Gutierrez de Pando, Hospital de Mataró (Spain)
Effrosyni Anastasiadou, Ippokrateio General Hospital, Thessaloniki (Greece)
Elisabeth Henrion, CHRSM site Meuse, Namur (France)
Elisabeth Kooi, UMCG (Netherlands)
Elisabeth Norman, Skåne University Hospital Lund (Sweden)
Elsa Kermorvant, Necker-Enfants malades Hospital (France)
Enrico Rosati, Azienda Ospedaliera Card. G. Panico – Tricase (Italy)
Enrique Criado, Hospital Clinico San Carlos (Spain)
Eric Cavatorta, CHU Charleroi (Belgium)
Estañ-Capell J, Hospital Clínico Universitario Valencia (Spain)
Falaina Vasiliki, General Hospital of Nikaia "Agios Panteleimon" (Greece)
Fasolato Valeria, Carlo Poma Mantova (Italy)

Federico Schena, AO SS. Antonio e Biagio e C.Arrigo – Alessandria (Italy)
Fermín García-Muñoz Rodrigo, Hospital Universitario Insular Materno-Infantil de Las Palmas (Spain)
Fernando Agama Cuenca, Hospital General Enrique Garcés (Ecuador)
Feroza Aktar, Chattogram maa shishu o general hospital medical College (Bangladesh)
Flamein, CHU Lille (France)
Flavia Pronzato Cuello, Hospital General Universitario de Castellón (Spain)
Francesco, University Hospital of Verona (Italy)
Funda Tuzun, Dokuz Eylül University Hospital (Turkey)
Gabriel Dimitriou, University General Hospital of Patras (Greece)
Gabriela Mimoso, Centro Hospitalar e Universitário de Coimbra (Portugal)
Gemma Arca, Hospital Clinic (Spain)
Georgios Mavrogeorgos, Aglaia Kyriakou Children's Hospital (Greece)
Gianluca Lista, V.Buzzi- Ospedale dei Bambini, Milan (Italy)
Gloria Cristofori, Fondazione IRCCS Ca' Granda Ospedale Maggiore Policlinico, Milan (Italy)
Guellec, CHU Nice, Hôpital L'Archet (France)
H. Gözde Kanmaz Kutman, University of Health Sciences, Ankara City Hospital (Turkey)
Hallvard Reigstad, Haukeland university hospital (Norway)
Hana Cihlarova, General University Hospital, 1st Faculty of Medicine Charles University, Prague (Czech Republic)
Hana Wiedermannová, Univerzity hospital Ostrava (Czech Republic)
Hector Boix, Hospital Dexeus (Spain)
Helmut Küster, University Medical Center Göttingen (Germany)
Henrique Soares, Centro Hospitalar Universitário de São João, Porto (Portugal)
Henrique Soares, Centro Hospitalar Universitário de São João, Porto (Portugal)
Hosu Anamaria, Alba Iulia Emergency County Hospital (Romania)
Ilya, KGBUZ AKKPC Barnaul (Russia)
Isabella Mauro, Azienda Sanitaria Universitaria Integrata di Udine (Italy)
Iulia Ercuta, Scuc Grigore Alexandrescu (Romania)
Jan Malý, Charles University, Faculty of Medicine and University Hospital, Hradec Králové (Czech Republic)
Jannicke Andresen, Oslo University hospital, Rikshospitalet (Norway)
Javier Miranda-Mallea, Hospital Vithas Valencia 9 de Octubre (Spain)
Jean-Claude Fauchere, University Hospital Zurich (Switzerland)
Jean-Michel Roue, Brest University Hospital (France)
Jesca Nakibuka, Kawempe National Referral Hospital (Uganda)
Jesper Fenger-Grøn, Lillebelt Hospital (Denmark)
Johanna Ivancsó, Borsod-Abauj-Zemplén County Hospital (Hungary)
Jose Luis Fernández Trisac, Hospital Teresa Herrera CHUAC A Coruña (Spain)
Jose Maria Lloreda Garcia, Hospital Santa Lucía de Cartagena (Spain)
Jovandaric Z. Miljana, University Clinical Centre of Serbia (Serbia)
Jozsef Korcsik, St. Vincenz Krankenhaus, Kinderklinik, Limburg an der Lahn (Germany)
Juan Calviño, CHTMAD (Portugal)
Judit Kiss, University of Szeged (Hungary)
Juliana Patkai, Port-Royal NICU, Cochin Hospital, Paris (France)
Juliane Schneider, Lausanne University hospital (Switzerland)
Katarina Matasova, Jessenius Faculty of Medicine in Martin, Comenius University, University Hospital Martin (Slovakia)
Katarzyna Wróblewska-Seniuk, Poznań University of Medical Sciences (Poland)
Katrin Klebermass-Schrehof, Medical University of Vienna (Austria)
Klaudia Demová, Faculty hospital Nove Zamky (Slovakia)
Kosmas Sarafidis, Aristotle University of Thessaloniki (Greece)
Kuhn Pierre, University Hospital of Strasbourg (France)
Kyla Marks, Soroka University Medical Centre (Israel)
Laura Castells Vilella, Hospital Universitari General de Catalunya Grupo Quirónsalud (Spain)
Laura Fazilleau, University Hospital of Caen (France)
Laura San Feliciano, Hospital universitario de Salamanca (Spain)
Laura Sánchez García, La Paz University Hospital (Spain)
Laura, Ospedale Filippo del Ponte, Varese (Italy)
Laureline Dubray, Centre Hospitalier Rives de Seine (France)
Lauterbach Ryszard, University Hospital Kraków (Poland)
Liam Mahoney, St. Michael's Hospital, Bristol (United Kingdom)
Linda Vad Pedersen, Aarhus University Hospital (Denmark)
Lise Aunsholt, Rigshospitalet (Denmark)
Luca, Bolzano Hospital (Italy)
Maissa Rayyan, University Hospitals Leuven (Belgium)
Manuel Bernal Benitez, Hospital de Especialidades Miguel Hidalgo (Mexico)
Marcelino Pumarada Prieto, Hospital Universitario Alvaro Cunqueiro (Spain)
Marcio Fossari, Unimed Litoral Maternity (Brazil)
Marcus Krueger, Munich Municipal Hospital Group (Germany)
Maria Francesca Campagnoli, S. Anna Hospital Turin (Italy)
Maria Stamatin, Cuza-Voda Clinical Hospital of Obstetrics and Gynecology (Romania)

Maria Taboada Perianes, CHUAC (Spain)
Marie Chevalier, CHU de Grenoble (France)
Mari-Liis Ilmoja, Tallinn Children’s Hospital (Estionia)
Marine Dorsi, CHT Noumea (France)
Marjo Metsäranta, Helsinki University Hospital (Finland)
Mark Dzietko, University Essen (Germany)
Mark Johnson, University Hospital Southampton NHS Foundation Trust (United Kingdom)
Mark Prutkin, Regional Children;s Hospital (Russia)
Marta Camprubi Camprubi, Hospital Sant Joan de Deu (Spain)
Marta Padín, Hospital Alvaro Cunqueiro (Spain)
Martin Wald, Uniklinikum Landeskrankenhaus Salzburg (Austria)
Martin Weissensteiner, Kepler Uniklinikum Linz (Austria)
Mateusz Jagła, Children's University Hospital, Jagiellonian University, Krakow (Poland)
Merazzi Daniele, Valduce Hospital (Italy)
Michael Fleischer, Klinikum Lippe Detmold (Germany)
Miryan Mateos Polo, Hospital Universitario de Salamanca (Spain)
Mohammed Gaffari, Wom en's Wellness and Research Center (Qatar)
Morten S. Lindhard, Randers Regional Hospital (Denmark)
Naveed Hussain, University of Connecticut (USA)
Nele Howold, Klinikum Wolfsburg (Germany)
Neli Jekova, University hospital "Maicin dom ", Sofia (Bulgaria)
Nicholas Embleton, Newcastle Hospitals NHS Trust (United Kingdom)
Nicola Laforgia, AOUC Policlinico Bari (Italy)
Niemarkt, Maxima Medical Centre Veldhoven (Netherlands)
Nigel Kennea, St George's Hospital London (United Kingdom)
Niketa Kolici, Amerikan Hospital 3 (Albania)
Nilgun Karadag, Zeynep Kamil Hospital, Istanbul (Turkey)
Nunzia Decembrino, AOU Policlinico G Rodolico San Marco Catania (Italy)
Nurdan Dinlen Fettah, Dr Sami Ulus Children’s Hospital (Turkey)
Nuria Torre, Parc Taulí (Spain)
Oleg Nikolaenko, Crimean Republic Child Clinical Hospital (Russia)
Olena, Перинатальный центр (Ukraine)
Olivier Claris, Hopital Femme Mère Enfant (France)
Olivier Danhaive, Saint-Luc University Hospital, Catholic University of Louvain, Brussels (Belgium)
Omer Erdeve, Ankara University Children's Hospital (Turkey)
Outi Tammela, Tampere University hospital (Finland)
Paola Lago, Cà Foncello Regional Hospital Treviso (Italy)
Paola Marcozzi, Azienda Ospedaliera San Camillo Forlanini (Italy)
Pasqua Betta, AOU Policlinico Rodolico Catania (Italy)
Patricia Alonso López, Hospital severo ochoa (Spain)
Pelluau, CHU Toulouse (France)
Pfister, HUG (Switzerland)
Ramful Duksha, CHU Felix Guyon, Saint-Denis de La Réunion (France)
Raquel Adsarias Ferrera, Hospital Universitario General de Catalunya (Spain)
Rasa Brinkis, Hospital of Lithuanian University of Health Sciences Kauno klinikos (Lithuania)
Roberto Bellù, Ospedale Mnazoni, Lecco (Italy)
Roberto Ortiz Movilla, Hospital Universitario Puerta de Hierro-Majadahonda (Spain)
Robin van der Lee, Amalia Children's Hospital Radboudumc Nijmegen (Netherlands)
Rocío López Ruiz, Hospital de Manises (Spain)
Roser Porta, Hospital Germans Trias i Pujol (Spain)
Ruth del Rio, Hospital Sant Joan de Déu (Spain)
Ruth Gottstein, St. Mary's Hospital, Manchester (United Kingdom)
Safiye Elif Uzlu, Ankara Etlik Zubeyde Hanim Egitim ve Arastirma Hastanesi (Turkey)
Salih Çağrı Çakır, Bursa Doruk Hospital (Turkey)
Sandra Salas Garcia, Hospital General Universitari Castelló (Spain)
Sanjeev Deshpande, Princess Royal Hospital, Telford (United Kingdom)
Sarah Aidibe Kadra, Clinique
Satyen K Hemrajani, Fortis Escorts Hospital, Jaipur (India)
Seda Yilmaz Semerci, Istanbul Kanuni Sultan Suleyman Training and Research Hospital (Turkey)
Senem Alkan, Behcet Uz Children's Hospital (Turkey)
Serafina Perrone, University Hospital of Parma (Italy)
Sergey V. Minaev, Pediatric Regional Clinical Hospital (Russia)
Sergey, Children’s hospital (Ukraine)
Sezin Unal, Health Sciences University Ankara Etlik Zübeyde Hanım Maternity Hospital (Turkey)
Shteryu Boyadzhiev, Lozenetz Hospital (Bulgaria)
Sideri Vasiliki, Attikon Hospital Chaidari-Athens (Greece)
Silke Haag, Diakonissen-Stiftungs-Krankenhaus Speyer (Germany)
Sithembiso Mbatha, Bheki Mlangeni District Hospital (South Africa)

Smeets, CHC Montlegia (Belgium)
Sopapan Ngerncham, Siriraj Hospital, Mahidol University, Bangkok (Thailand)
Sophie Vanhaesebrouck, UZ Gent (Belgium)
Srinivasarao Babarao, Liverpool Women's Hospital (United Kingdom)
Stefan Johansson, Sachs Children’s Hospital (Sweden)
Stefano Ghirardello, Fondazione IRCCS policlinico San Matteo Pavia (Italy)
Stocker Martin, Children’s Hospital Lucerne (Switzerland)
Thomas Kühn, Vivantes Berlin (Germany)
Thomas Strahleck, Klinikum Stuttgart Olgahospital (Germany)
Thordur Thorkelsson, Children's Hospital Iceland (Iceland)
Timo Saarela, Oulu University Hospital (Finland)
Tomas Juren, Faculty Hospital Brno (Czech Republic)
Tomás Sánchez Tamayo, Hospital Regional Universitario de Málaga (Spain)
Tuuli Metsvaht, Tartu University hospital (Estonia)
Twan Mulder, University Hospital Antwerp (Belgium)
Ulla Sankilampi, Kuopio University Hospital (Finland)
Urlesberger, Medical University of Graz (Austria)
Valentina, Umberto I Siracusa (Italy)
Victoria Ramos Ramos, Hospital Universitario de Jerez (Spain)
Victoria, Hospital Central Dr Ignacio Motines Prieto (Mexico)
Vincent Rigo, CHR Citadelle Liège (Belgium)
Vincenzo Salvo, Giovanni Paolo II Hospital, Ragusa (Italy)
Vladan Milovanov, Hospital of Southwestern Jutland (Denmark)
W Boehm, Centre Hospitalier de Luxembourg (Luxembourg)
Xavier Durrmeyer, Centre Hospitalier Intercommunal de Créteil (France)
Yolanda Ruiz del Prado, Hospital San Pedro Logroño La Rioja (Spain)
Zana-Taieb, Hôpital Cochin Port Royal (France)
Zbynek Stranak, Institute for the Care of Mother and Child (Czech Republic)
Zeynep Alp Unkar, Istanbul University-Cerrahpasa, Cerrahpasa Faculty of Medicine (Turkey)
Ольга Иванова, Иркутский городской перинатальный центр (Russia)

Supplementary Material 2. Overview of the questionnaire

Section	Question	Question type	Answer options
NICU demographics	Where do you work? [m]	MC	List of European countries
	In which hospital do you work? [m]	OQ	Free text
	How would you classify the level of care in your neonatology unit? [m]	CQ	Level I (well newborn nursery; basic level of care to low-risk neonates)<br>Level II (special care nursery; care for stable or moderately ill neonates)<br>Level III (neonatal intensive care unit; care for infants who are born very preterm or with very low birth weight)<br>Level IV (referral NICU; care for critically ill neonates, pediatric surgical consultants available 24/7)
	What is your function? [m]	MC	Neonatologist<br>Pediatrician<br>Other: …
	How many neonates were approximately admitted to your NICU in 2020?	OQ	Free text
	How many neonates with a birth weight below 1500 grams were approximately admitted to your NICU in 2020?	OQ	Free text
	How many patients with necrotizing enterocolitis are approximately treated in your hospital yearly?	MC	0<br>1-10<br>10-20<br>20-30<br>30-40<br>40-50<br>>50<br>I don't know
Protocols for analgesic therapy	Does your hospital have a written standard protocol for analgesic therapy in neonates? [m]	MC	Yes<br>No<br>I don't know
	Does your protocol include clear definitions of indications for starting, adjusting and stopping analgesics? [m, c]	MC	Yes<br>No<br>I don't know
	Does your hospital have a specific protocol for analgesic therapy in NEC patients? [m, c]	MC	Yes<br>No<br>I don't know
	How would you describe the level of adherence to this protocol in your center? [c]	MC	Very high (80-100% of cases)<br>High (60-80% of cases)<br>Intermediate (40-60% of cases)<br>Low (20-40% of cases)<br>Very low (0-20% of cases)
Pain measurement	Is a NEC patient's pain level measured in your NICU? [m]	MC	Yes<br>No
	Which method(s) is/are used to assess pain in your NICU? [m]	CQ	COMFORTneo score<br>Numerical Rating Scale (NRS)<br>Neonatal Infant Pain Scale (NIPS)<br>Neonatal Pain, Agitation, and Sedation Scale (N-PASS)<br>Premature Infant Pain Profile (-Revised) (PIPP(-R))<br>Échelle de la Douler Inconfort Nouveau-Né (EDIN)<br>Cries, Requires oxygen, Increased vital signs, Expression, Sleeplessness (CRIES)<br>Neonatal Facial Coding System (NFCS)<br>Bernese Pain Scale for Neonates (BPSN)<br>Other: …
	Who assess a patient's pain level in your NICU?	CQ	Nurses<br>Physicians<br>Parents<br>Other: …

	How often is a NEC patient's pain level assessed in your NICU? [m]	CQ	1-2 times a day<br>3-4 times a day<br>5-6 times a day<br>>6 times a day<br>On indication<br>Other: …
Analgesic therapy for NEC patients	Is sucrose used to treat pain in NEC patients who are nil per os (NPO) in your NICU?	MC	Yes<br>No
	Are any other non-pharmacological interventions used to treat pain in NEC patients? If yes, which non-pharmacological intervention(s)?	MC/OQ	Yes: …<br>No
	Are NEC patients in your NICU ever treated with epidural analgesics?	MC	Yes<br>No
	Are NEC patients in your NICU ever treated with intravenous analgesics? [m]	MC	Yes<br>No
	When is treatment with intravenous analgesics started? [m, c]	MC	Pre-emptive<br>On indication (in case of pain)<br>Other: …
	Which analgosedative or which combination of analgosedatives is used to start treating a NEC patient in your NICU? [m, c]	CQ	Morphine<br>Fentanyl<br>Methadone<br>Paracetamol<br>Ketamine<br>Tramadol<br>Midazolam<br>Sufentanil<br>Other: …
	Is [selected analgosedative] administered continuously, intermittently (bolus medication) or both? [m, c]	MC	Continuously<br>Intermittently (bolus medication)<br>Continuously + intermittenly
	Are loading doses used before continuous [selected analgosedative] infusion in NEC patients in your NICU? [m, c]	MC	Yes<br>No
	Which doses of continuous [selected analgosedative] are used to treat NEC patients in your NICU? [c]	OQ	Lower limit / Upper limit<br>Loading dose (ug/kg): … / …<br>Maintenance dose (ug/kg/h): … / …
	Which doses of intermittent [selected analgosedative] are used to treat NEC patients in your NICU? [c]	OQ	Lower limit / Upper limit<br>Bolus dose (ug/kg): … / …<br>Number of boluses per day: … / …
	Are any analgosedatives contra-indicated for treatment in NEC patients in your NICU? If yes, which? [c]	MC/OQ	Yes: …<br>No
	Does analgosedative treatment in NEC patients in your NICU differ for ventilated and non-ventilated patients? If yes, in what way does it differ? [c]	MC/OQ	Yes: …<br>No
	How is analgosedative treatment in your NICU intensified when the patient experiences pain under the initial analgesic therapy? [m, c]	CQ	Increase the dose of the current analgesic(s)<br>Add another analgesic to the current analgesic(s)<br>Switch the current analgesic(s)<br>Other: …
	Of which analgosedative(s) is the dose increased? [c]	CQ	The analgosedatives selected previously in the question "Which analgosedatives or which combination of analgosedatives is used to start treating a NEC patient in your center?"
	Which analgosedative(s) is/are added? [c]	CQ	Same list of options as in the question "Which analgosedatives or which combination of analgosedatives is used to start treating a NEC patient in your center?" minus the options that were selected in this previous question
	Which analgosedative(s) is/are switched? [c]	CQ	The analgosedatives selected previously in the question "Which analgosedatives or which combination of

			analgosedatives is used to start treating a NEC patient in your center?”
	To which analgosedative(s) is switched? [c]	CQ	Same list of options as in the question “Which analgosedatives or which combination of analgosedatives is used to start treating a NEC patient in your center?” minus the options that were selected in this previous question
	Is [selected analgosedative] administered continuously, intermittently (bolus medication) or both? [m, c]	MC	Continuously<br>Intermittently (bolus medication)<br>Continuously + intermittently
	Are loading doses used before continuous [selected analgosedative] infusion in NEC patients in your NICU? [m, c]	MC	Yes<br>No
	Which doses of continuous [selected analgosedative] are used to treat NEC patients in your NICU? [c]	OQ	Lower limit / Upper limit<br>Loading dose (ug/kg) … …<br>Maintenance dose (ug/kg/h) … …
	Which doses of intermittent [selected analgosedative] are used to treat NEC patients in your NICU? [c]	OQ	Lower limit / Upper limit<br>Bolus dose (ug/kg) … …<br>Number of boluses per day … …
Your opinion on pain management in your NICU	In your opinion, does the pain measurement instrument you use adequately measure pain in NEC patients? [c]	MC	Yes<br>No
	Do you think the current analgesic therapy regimen for NEC patients in your NICU is adequate?	MC	Yes<br>No
	How do you think the current pain management for NEC patients in your NICU could be improved?	OQ	Free text

[m] This question is mandatory.

[c] This question is shown conditionally on the answer to a previous question.

MC = multiple choice; CQ = checkbox question (i.e. multiple options can be chosen); OQ = open question

Supplementary Material 3. Most used pain measurement instrument per country and the percentage of NICUs using this instrument

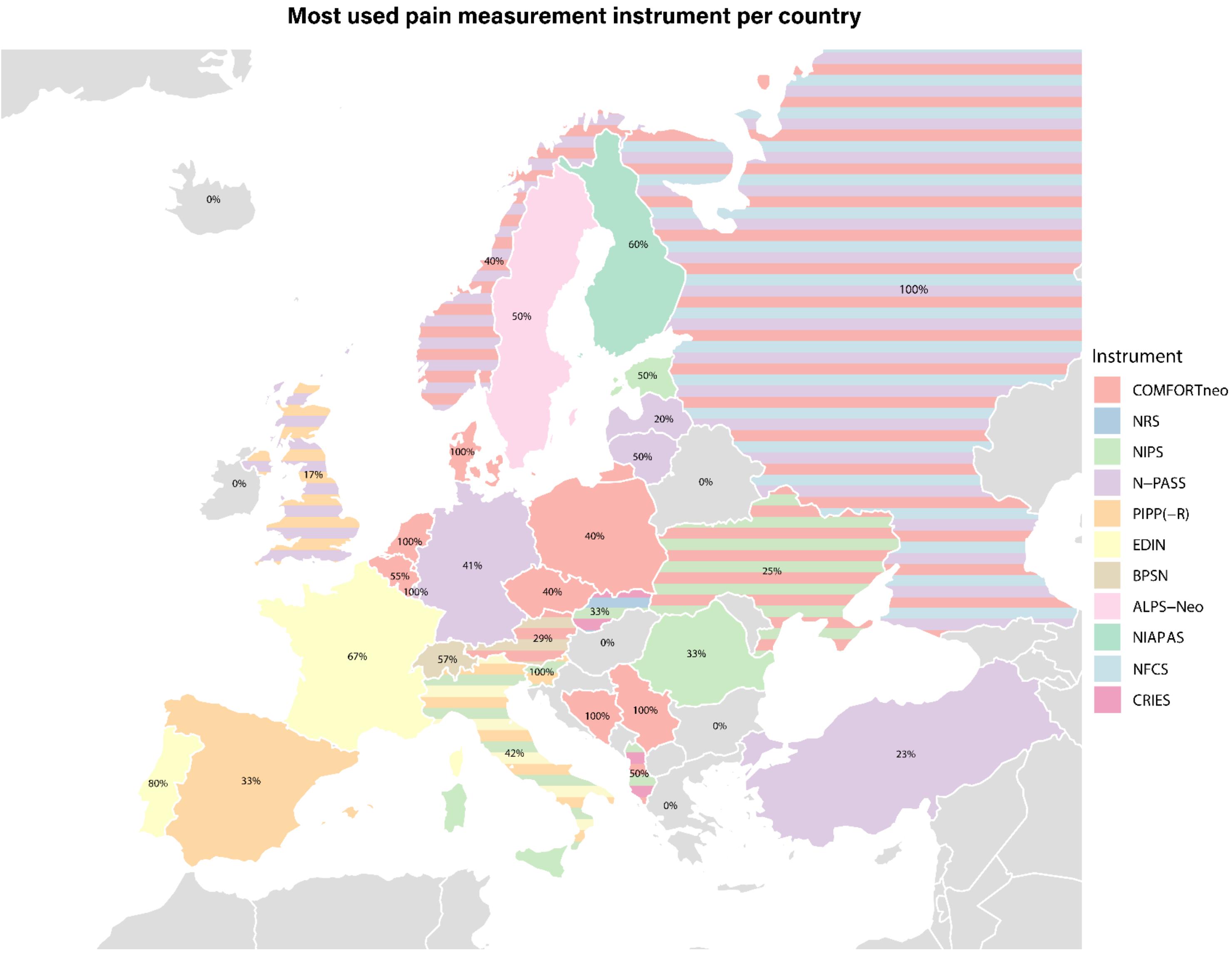

In some countries, there were two or three most used analgosedatives which were used equally often. This has been indicated with a striped pattern.

Abbreviations: NRS, Numerical Rating Scale; NIPS, Neonatal Infant Pain Scale; N-PASS, Neonatal Pain, Agitation and Sedation Scale; PIPP(-R), Premature Infant Pain Profile (-Revised); EDIN, Échelle Douleur Inconfort Nouveau-né; BPSN, Bernese Pain Scale for Neonates; ALPS-Neo, Astrid Lindgren and Lund Children's Hospitals Pain and Stress Assessment Scale for Preterm and sick Newborn Infants; NIAPAS, Neonatal Infant Acute Pain Assessment Scale; NFCS, Neonatal Facial Coding System; CRIES, Crying Requires oxygen Increased vital signs Expression Sleep

Supplementary Material 4. Pain management practices for NEC in European NICUs (n=244)

Variable	
Sucrose used for NPO NEC patients	
Yes	96 (39.3)
No	119 (48.8)
Missing	29 (11.9)
Non-pharmacological interventions used	
Yes	141 (57.8)
No	103 (42.2)
Type of non-pharmacological interventions used	
Containment (e.g., facilitated tucking, swaddling, nesting, cocooning)	86 (35.2)
Touch (e.g., holding, cuddling, kangaroo care)	45 (18.4)
Non-nutritive sucking (pacifier)	43 (17.6)
Positioning	22 (9.0)
Parental involvement/voice	12 (4.9)
Maternal milk (e.g., feeding, odor diffuser)	10 (4.1)
Low lights and quietness	9 (3.7)
NIDCAP care	8 (3.3)
Minimal handling	7 (2.9)
Music	5 (2.0)
Glucose or dextrose	4 (1.6)
Other (e.g., nursing, sensorial saturation, gastric tube, massage)	13 (5.3)
Epidural analgesics used for NEC patients	
Yes	5 (2.0)
No	219 (89.8)
Missing	20 (8.2)
Intravenous analgesics used for NEC patients	
Yes	225 (92.2)
No	19 (7.8)

Values are expressed as number of NICUs (%)
NPO = nil per os, i.e., withholding oral food and fluids

Supplementary Material 5. Most used analgosedative per country and the percentage of NICUs administering this analgosedative

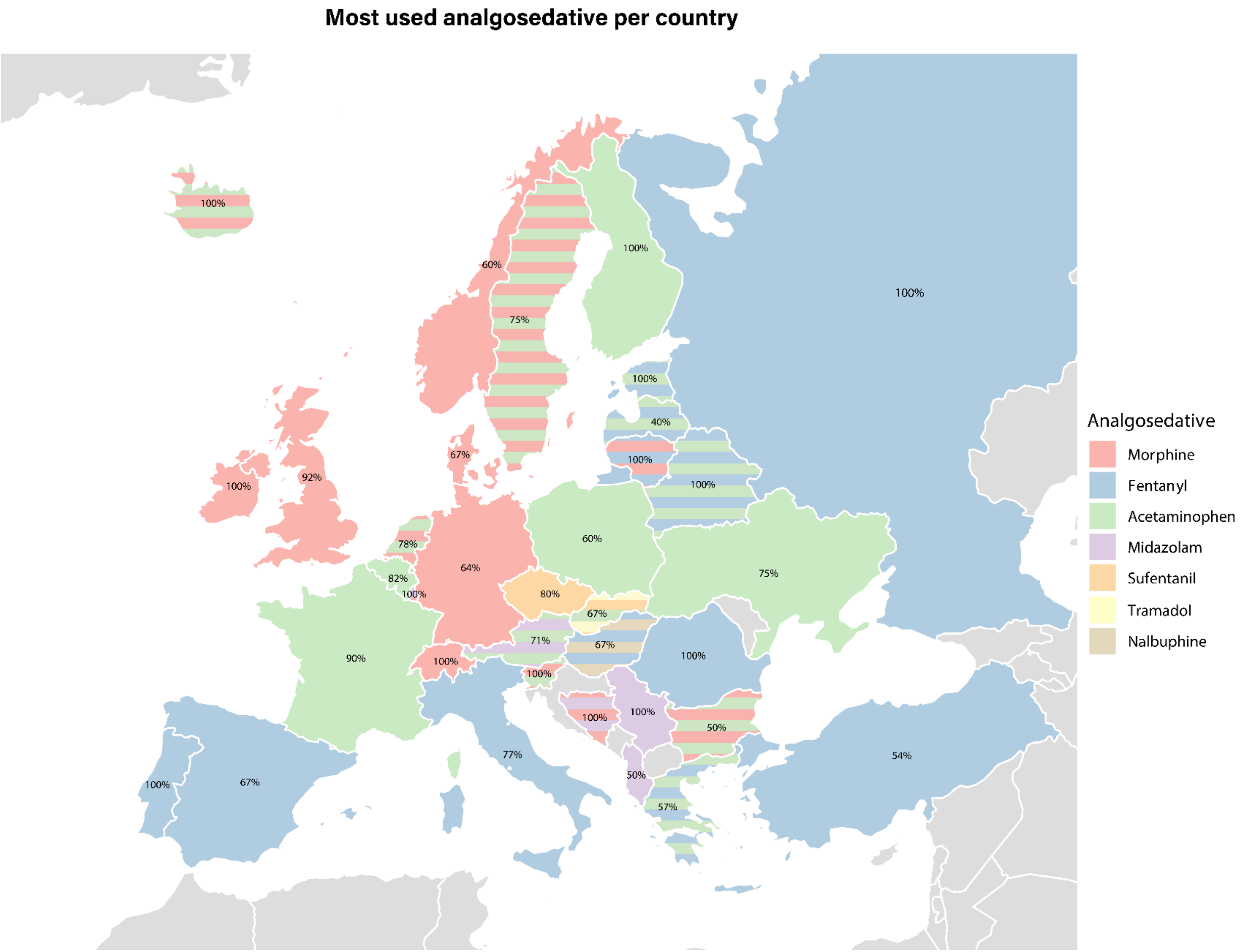

In some countries, there were two or three most used analgosedatives which were used equally often. This has been indicated with a striped pattern.

Supplementary Material 6. Dose ranges of prescribed analgosedatives

Analgosedative	Lower limit	Upper limit
Acetaminophen		
Bolus dose (mg/kg)	10 (7.5-10)	15 (10-15)
Boluses per day	3 (2-4)	4 (4-4)
Fentanyl		
Loading dose (ug/kg)	1.0 (1.0-2.0)	3.0 (2.0-4.0)
Maintenance dose (ug/kg/h)	1.0 (0.5-1.0)	3.0 (3.0-5.0)
Bolus dose (ug/kg)	1.0 (0.9-1.0)	2.0 (2.0-3.0)
Boluses per day	2 (1-4)	6 (4-8)
Morphine		
Loading dose (ug/kg)	50 (10-50)	100 (50-100)
Maintenance dose (ug/kg/h)	10 (5-10)	40 (20-50)
Bolus dose (ug/kg)	25 (10-50)	100 (30-100)
Boluses per day	2 (1-4)	6 (4-8)
Midazolam		
Loading dose (mg/kg)	0.05 (0.05-0.10)	0.15 (0.10-0.20)
Maintenance dose (mg/kg/h)	0.04 (0.01-0.10)	0.20 (0.06-0.40)
Bolus dose (mg/kg)	0.05 (0.05-0.10)	0.18 (0.10-0.20)
Boluses per dayswit	1 (1-4)	6 (4-10)
Sufentanil		
Loading dose (ug/kg)	0.10 (0.10-0.20)	0.30 (0.29-0.50)
Maintenance dose (ug/kg/h)	0.10 (0.10-0.20)	0.60 (0.40-1.88)
Bolus dose (ug/kg)	0.10 (0.10-0.10)	0.50 (0.23-0.88)
Boluses per day	2 (1-2)	8 (6-8)
Sufentanil		
Loading dose (ug/kg)	0.10 (0.10-0.20)	0.30 (0.29-0.50)
Maintenance dose (ug/kg/h)	0.10 (0.10-0.20)	0.60 (0.40-1.43)
Bolus dose (ug/kg)	0.10 (0.10-0.10)	0.50 (0.23-0.88)
Boluses per day	2 (1-2)	8 (6-8)
Ketamine		
Loading dose (mg/kg)	0.75 (0.50-1.00)	1.25 (1.00-1.88)
Maintenance dose (mg/kg/h)	0.30 (0.10-1.00)	1.00 (0.50-2.00)
Bolus dose (mg/kg)	1.00 (0.50-1.00)	2.00 (1.13-2.00)
Boluses per day	1 (1-2)	5 (4-6)
Nalbuphine		
Loading dose (mg/kg)	0.10 (0.10-0.10)	0.20 (0.20-0.20)
Maintenance dose (mg/kg/h)	0.008 (0.008-0.008)	0.05 (0.05-0.05)
Bolus dose (mg/kg)	0.10 (0.06-0.10)	0.20 (0.12-0.23)
Boluses per day	2 (1-3)	4 (4-6)
Tramadol		
Loading dose (mg/kg)	1.50 (1.25-1.75)	2.50 (2.25-2.75)
Maintenance dose (mg/kg/h)	2.00 (1.50-4.00)	3.00 (2.50-6.50)
Bolus dose (mg/kg)	1.50 (1.25-1.75)	2.00 (2.00-2.00)
Boluses per day	4 (4-4)	7 (7-8)
Clonidine		
Loading dose (mg/kg)	1.00 (1.00-1.00)	2.00 (2.00-2.00)
Maintenance dose (mg/kg/h)	0.30 (0.20-2.65)	0.50 (0.40-1.25)
Dexmedetomidine		
Maintenance dose (mg/kg/h)	0.20 (0.05-0.28)	1.00 (0.25-1.30)
Methadone		
Bolus dose (ug/kg)	25 (0.05-50)	50 (0.15-200)
Boluses per day	2 (1-3)	5 (4-6)
Remifentanil		
Maintenance dose (mg/kg/h)	0.50 (0.25-2.75)	2.00 (1.00-16.00)
Metamizole		
Bolus dose (mg/kg)	10.0 (10.0-15.0)	20.0 (17.5-30.0)
Boluses per day	3 (2-3)	4 (4-5)
Ketofol		
Loading dose (mg/kg)	1.50 (1.50-1.50)	3.00 (3.00-3.00)
Maintenance dose (mg/kg/h)	0.30 (0.30-0.30)	0.50 (0.50-0.50)
Bolus dose (mg/kg)	0.40 (0.30-0.30)	3.00 (3.00-3.00)
Boluses per day	3 (3-3)	6 (6-6)
Piritramide		
Bolus dose (mg/kg)	0.05 (0.05-0.05)	0.10 (0.10-0.10)
Boluses per day	1 (1-1)	3 (3-3)
Pethidine		
Bolus dose (mg/kg)	0.05 (0.05-0.05)	0.10 (0.10-0.10)
Boluses per day	1 (1-1)	8 (8-8)

Values are expressed as median (IQR)

Supplementary Material 7. Overview of intensified analgesic therapy strategies

**Variable**	
How is analgosedative treatment intensified?	
Increase the dose of the current analgosedative(s)	207 (92.0)
Add another analgosedative	120 (53.3)
Switch to another analgosedative	51 (22.7)
Other	7 (3.1)
Of which analgosedative is the dose increased?	
Fentanyl	109 (48.8)
Morphine	95 (42.2)
Midazolam	22 (9.8)
Sufentanil	22 (9.8)
Acetaminophen	19 (8.4)
Ketamine	9 (4.0)
Nalbuphine	5 (2.2)
Tramadol	3 (1.3)
Clonidine	3 (1.3)
Dexmedetomidine	3 (1.3)
Methadone	2 (0.9)
Remifentanil	2 (0.9)
Metamizole	1 (0.4)
Oxycodone	1 (0.4)
Piritramide	1 (0.4)
Pethidine	1 (0.4)
Which analgosedative is added?	
Midazolam	29 (12.9)
Ketamine	25 (11.1)
Dexmedetomidine	17 (7.6)
Fentanyl	16 (7.1)
Acetaminophen	16 (7.1)
Morphine	10 (4.4)
Tramadol	5 (2.2)
Methadone	4 (1.8)
Sufentanil	4 (1.8)
Clonidine	3 (1.3)
Diazepam	2 (0.9)
Phenobarbital	2 (0.9)
Remifentanil	1 (0.4)
Metamizole	1 (0.4)
Which analgosedative is switched (from)?	
Morphine	28 (12.4)
Fentanyl	18 (8.0)
Acetaminophen	6 (2.7)
Midazolam	4 (1.8)
Nalbuphine	2 (0.9)
Tramadol	1 (0.4)
Sufentanil	1 (0.4)
Clonidine	1 (0.4)
Metamizole	1 (0.4)
Oxycodone	1 (0.4)
To which analgosedative is switched?	
Fentanyl	10 (4.4)
Morphine	8 (3.6)
Sufentanil	6 (2.7)
Ketamine	4 (1.8)
Methadone	2 (0.9)
Tramadol	2 (0.9)
Midazolam	2 (0.9)
Dexmedetomidine	2 (0.9)
Hydromorphone	1 (0.4)

Values are expressed as number of NICUs (%)

Supplementary Material 8. Number of responses from countries outside Europe

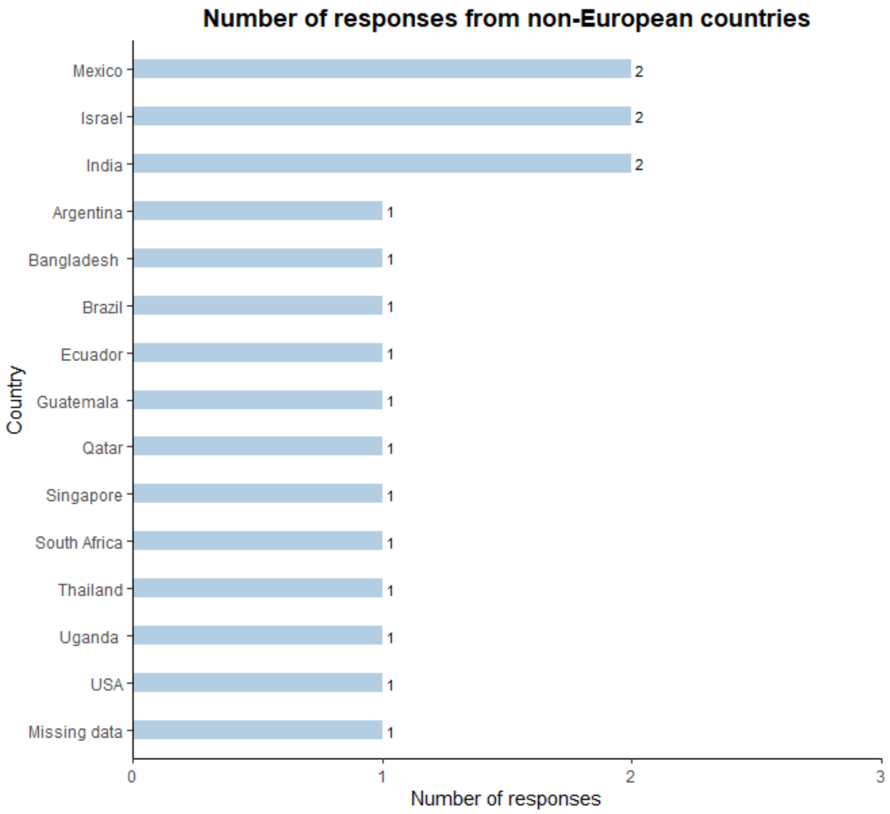

Supplement: Supplementary file 1 — Supplementary Material 1 [file 41390_2023_2508_MOESM1_ESM.pdf]
